# Addressing the Clinical Feasibility of Adopting Circulating miRNA for Breast Cancer Detection, Monitoring and Management with Artificial Intelligence and Machine Learning Platforms

**DOI:** 10.3390/ijms232315382

**Published:** 2022-12-06

**Authors:** Lloyd Ling, Ahmed Faris Aldoghachi, Zhi Xiong Chong, Wan Yong Ho, Swee Keong Yeap, Ren Jie Chin, Eugene Zhen Xiang Soo, Jen Feng Khor, Yoke Leng Yong, Joan Lucille Ling, Naing Soe Yan, Alan Han Kiat Ong

**Affiliations:** 1 Lee Kong Chian Faculty of Engineering & Science, Universiti Tunku Abdul Rahman, Kajang 43000, Malaysia; 2 M. Kandiah Faculty of Medicine and Health Sciences, Universiti Tunku Abdul Rahman, Kajang 43000, Malaysia; 3 Division of Biomedical Sciences, School of Pharmacy, Faculty of Sciences and Engineering, University of Nottingham Malaysia, Semenyih 43500, Malaysia; 4 China-ASEAN College of Marine Sciences, Xiamen University Malaysia, Sepang 43900, Malaysia; 5 Department of Computing and Information Systems, Sunway University, Petaling Jaya 47500, Malaysia; 6 Edson College of Nursing and Health Innovation, Arizona State University, Phoenix, AZ 85004, USA

**Keywords:** liquid biopsy, circulating miRNA, breast cancer, AI, ML

## Abstract

Detecting breast cancer (BC) at the initial stages of progression has always been regarded as a lifesaving intervention. With modern technology, extensive studies have unraveled the complexity of BC, but the current standard practice of early breast cancer screening and clinical management of cancer progression is still heavily dependent on tissue biopsies, which are invasive and limited in capturing definitive cancer signatures for more comprehensive applications to improve outcomes in BC care and treatments. In recent years, reviews and studies have shown that liquid biopsies in the form of blood, containing free circulating and exosomal microRNAs (miRNAs), have become increasingly evident as a potential minimally invasive alternative to tissue biopsy or as a complement to biomarkers in assessing and classifying BC. As such, in this review, the potential of miRNAs as the key BC signatures in liquid biopsy are addressed, including the role of artificial intelligence (AI) and machine learning platforms (ML), in capitalizing on the big data of miRNA for a more comprehensive assessment of the cancer, leading to practical clinical utility in BC management.

## 1. Introduction

Globally, breast cancer (BC) remains the most common cancer and the leading cause of cancer death for women [1]. Despite the advancement in BC screening, diagnostics and therapy, the overall rates of BC incidence and mortality around the world have been on an increasing trend. Although BC mortality rates have declined over time in most high-income countries (HICs) [2], they remain high and are increasing in many low-middle-income and low-income countries [3,4], partly due to poor awareness and perception of early BC detection, leading to delays in diagnosis and treatment [1]. Recent studies show that the mortality rate is also compounded by disparities in BC screening between rural and urban rural/urban areas [5] as well as among those from different socio-economic and ethnic backgrounds of HICs [2]. Nevertheless, there has been a more established understanding of the heterogenous nature of BC and its application in the development of personalized medicine and targeted therapy [6]. However, the complex interaction within the tumor microenvironment (TME) and the influence of cancer stem cells (CSCs) from cancer recurrence to drug resistance [7] as well as population-based variation in terms of immunological response [8] continue to pose challenges in deciphering feasible approaches in managing BC diagnosis, monitoring and administrating effective treatment options. In the past decade, the evolution of BC diagnosis and classification has resulted in greater supporting tools, ranging from classical mammography and histopathology [9] to molecular-based markers and multigene prognosticators [10], which ultimately guided the overall management of BC (Table 1) [11,12,13,14]. Still, many if not all of these existing tools are heavily reliant on invasive tissue biopsies as the starting point for screening and monitoring BC progression, evaluating cancer prognosis and deciding on the best therapeutic options. Moreover, studies have shown that the BC signatures are often not clearly manifested and well represented in the current screening methods, namely mammography [15] and tissue biopsies [16]—more so in the diagnosis of metastatic cancers [17]. Liquid biopsy (LB), on the other hand, has emerged as a potential feasible approach in overcoming these shortfalls, from obtaining samples in a noninvasive manner to early detection of cancer and more comprehensive monitoring [18,19], thereby offering patients less stressful experiences and a better sense of worthiness in managing cancer treatments. Among the commonly known cancer biomarkers in LB [19], circulating microRNAs (miRNAs) stand out as a feasible and practical option [20]. Hence, this review will address the role of miRNA as a feasible candidate for liquid biopsy, not only in personalized BC management and targeted therapy but also in consolidating the big data of miRNA with artificial intelligence (AI) and machine learning platforms (ML) for a more comprehensive and inclusive approach in promoting effective BC patient care and outcome.

## 2. miRNA as Liquid Biopsy in Personalized Breast Cancer Management and Targeted Therapy

The liquid biopsy (LB) approach enables the securing of essential information on cancer progression and tumor through simple body-fluid-based samples, mainly through routine blood sampling. As gene-regulatory molecules in the body, circulating microRNAs (miRNAs) may be readily detected in plasma or serum of blood samples, enabling measurable changes in their levels, which are associated with the various conditions of the body, including cancers. Moreover, with the rapid advancement in bioinformatics in molecular data analysis, the inference of miRNAs with oncogene targets, cancer signaling pathways, survival analysis, prognostic values and drug targets is commonly obtainable [21] with cross-validation from clinical cancer-associated databases [22,23]. Although the application of miRNA as LBs for BC in the clinical setting is fairly new, with only two clinical studies recorded in www.clinicaltrials.gov as of October 2022 (Table 2), its utility may complement well with the existing standard clinical approach (Table 1), enhance diagnosis and monitoring of BC progression, as well as the response to treatments.

## 3. Current Trends and Research Outcomes of Circulating miRNA as Liquid Biopsy

Circulating miRNAs are extracellular miRNAs that are present in body fluids, such as blood, serum, plasma, milk, saliva and urine, either in the form of free-circulating miRNAs or encapsulated within extracellular vesicles (EVs), such as exosomes [24,25,26]. The advancement in molecular biology techniques has allowed scientists to employ different methods, such as real-time polymerase chain reaction (qPCR) [27], miRNA-sequencing (miRNA-seq) [28] and microarray [29], to detect the levels of circulating miRNAs among BC patients. These techniques have been reported to play essential roles in the diagnosis, classification and prognosis of BC [30,31]. We hope to provide an updated overview of the research findings that reported the association of circulating miRNAs to the diagnosis and prognosis of BC. Notably, EV-derived and exosomal miRNAs have attracted remarkable interest due to their superior stability to those of free-circulating miRNAs. As such, a separate cluster of studies that researched specifically miRNAs derived from EVs is also highlighted in this review.

### 3.1. Diagnostic Significance of Circulating miRNAs in Human Breast Cancer

To date, more than 50 original research articles have reported the roles of circulating miRNAs as a minimally invasive tool in diagnosing BC, either as free-circulating miRNA BC in plasma or serum (Table 3) [32,33] or transported in EVs and exosomes (Table 4) [34,35]. Many circulating miRNAs were shown to be elevated in BC patients as compared to healthy controls and these include miR-21 [36], miR-24 [32], miR-34 [37], miR-155 [38], miR-140-5p [39] and many other miRNAs [40]. Similarly, the downregulation of specific circulating miRNAs, such as miR-363-5p [25] and miR-4488 [41], was demonstrated to be useful to distinguish between BC patients and healthy controls. In addition, the elevation of serum exosomal miR-21-5p and miR-23a-3p levels [33] and plasma miR-133a, miR-148b and miR-200 levels [42,43] was found to be sensitive to distinguish early and advanced BC and this is particularly useful to detect early BC. Other miRNAs that had been reported to be useful in detecting early BC include miR-23b, miR-26b-5p, miR-106b-5p, miR-127-3p, miR-142-3p, miR-142-5p, miR-148b, miR-185-5p, miR-362-5p, miR-409-3p, miR-652 and miR-801 [44,45,46].

On the contrary, the level of circulating miRNAs is helpful to classify BC based on the clinical and histopathological grading [47,48]. For instance, the increased levels of exosomal miR-363-5p [25] and circulating miR-106a levels [49] were useful to differentiate BC with and without lymph node involvement. A clinical study reported that the elevation of four serum miRNAs, including miR-16-5p, miR-17-3p, miR-451a and miR-940, was observed in metastatic BC as compared to local, non-metastatic BC [50] and downregulation of plasma miR-195 level was also observed in metastatic BC [51]. The combined findings from these studies [50,51] highlighted the significance of circulating miRNA levels in BC staging. In addition, upregulation of plasma exosomal miR-181-5p and miR-222-3p is reported to link to advanced inflammatory BC as compared to non-inflammatory BC [35]. In terms of molecular grading, circulating miR-182 and miR-200c were shown to be downregulated and upregulated, respectively, in estrogen receptor (ER)- and progesterone receptor (PR)-positive patients. Upregulation of miR-10b and miR-21 was reported in ER-negative BC according to an Irish study [52], whereas suppression of miR-17 was shown to be observed in ER-positive BC [24]. For human epidermal growth factor receptor 2 (HER2)-positive patients, circulating miR-373 was reported to be upregulated [24] and, in another Chinese study [53], it was shown that the circulating level of miR-106a-5p and miR-20b-5p was increased in HER2-negative BC patients. In triple-negative BC (TNBC), serum miR-335 was reported to be downregulated [54], whereas serum miR-200c was upregulated [55]. Other circulating miRNAs that were upregulated in TNBC include miR-188-5p, miR-642b-3p, miR-1202, miR-1207-5p, miR-1225-5p, miR-1290, miR-3141, miR-4270 and miR-4281 [56].

### 3.2. Prognostic Significance of Circulating miRNAs in Human Breast Cancer

Apart from being employed as minimally invasive biomarkers in diagnosing and classifying different stages or types of BC (Table 3 and Table 4) [57], circulating miRNAs are also important in predicting the prognosis and treatment responses of BC patients [38,58]. For instance, the expression of miR-155 and miR-1246 was elevated in the plasma exosomes isolated from BC patients, and the upregulation of both miRNA levels was linked to poor survival, recurrence and trastuzumab resistance among BC patients [38]. In another study [39], downregulation of plasma miR-140-5p was correlated to increased chemoresistance, reduced event-free survival (EFS) and increased recurrence among BC patients. On the other hand, upregulation of exosomal miR-21 [58], exosomal miR-34a, miR-182 and miR-183 levels [59] was shown to contribute to poor chemotherapy response among BC patients, while the elevation of blood exosomal miR-2392, miR-4448 and miR-4800-3p was demonstrated to correlate to good response after neoadjuvant chemotherapy [60]. In terms of response towards targeted therapy, such as trastuzumab, decreased serum levels of miR-16-5p, miR-17-3p, miR-451a and miR-940 were observed in trastuzumab-resistant BC and the increased expression of these miRNAs was shown to promote treatment response to trastuzumab and improve survival among BC patients [50]. In another Arabic study [30], dysregulations of seven circulating miRNAs that include miR-19a, miR-19b-3p, miR-22-3p, miR-25-3p, miR-93-5p, miR-199a-3p and miR-210-3p were shown to be related to resistance to both chemotherapy and targeted therapy. On the contrary, the upregulation of circulating miR-21 correlated tightly to radio resistance among BC patients [36]. Notably, all the circulating miRNAs that were reported to modulate treatment responses in BC patients appeared to share a common function in promoting uncontrolled proliferation and apoptosis evasion among the BC cells [61,62]. For example, miR-373 was reported to upregulate the expression of vascular endothelial growth factor (VEGF) in BC cells, which would lead to enhanced proliferation and angiogenesis [62].

By studying the relationships between circulating miRNA levels, clinical conditions and treatment responses, clinicians could predict the survival and likelihood of disease recurrence among BC patients [63]. Elevation of several circulating exosomal miRNAs was shown to be associated with good survival and it was suggested that the upregulation of these miRNAs may improve patient survival by enhancing patient response to chemotherapy [60]. On the other hand, the upregulation of exosomal miR-200c [64] and miR-24-3p [47] was shown to correlate to poor overall survival (OS) among BC patients, as the increased expressions of these miRNAs were hypothesized to directly correlate to advanced disease staging [47,64]. Similarly, the downregulation of several circulating miRNAs, such as miR-34a [37] and miR-335 [54], was shown to reduce BC patient survival and this would contribute to an increased likelihood of relapse and recurrence. Metastasis is one of the important factors causing BC recurrence and the dysregulations. Eight miRNAs, including miR-296-3p, miR-575, miR-3610-5p, miR-4483, miR-4710, miR-4755-3p, miR-5698 and miR-8089, were demonstrated to be able to predict the likelihood of recurrence secondary to BC metastasis [31]. Other circulating miRNAs that are reported to play vital roles in influencing patient survival include miR-17, miR-18b, miR-103, miR-107, miR-652, miR-26b-5p, miR-106b-5p, miR-142-3p, miR-142-5p, miR-185-5p and miR-362-5p [45,65,66]. Patients with dysregulated circulating levels of these miRNAs were found to have more advanced disease staging and were more prone to face disease relapse and recurrence with reduced survival rate [45,65,66]. In two other studies [29,31], at least 20 circulating or exosomal miRNAs were reported to be sensitive and useful in distinguishing between recurrent and non-recurrent BC cases and this is helpful to predict patient prognosis and survival.

**Table 3:** Potential free-circulating miRNAs in diagnosing and predicting the prognosis or treatment responses among breast cancer patients. ↑ increase; ↓ reduce.

miRNAs	miRNAs Source	Diagnostic Significance	Significance in Grading/Classification	Prognostic Significance			Ref.
				Response to Treatment	Overall Survival	Relapse/Recurrence	
miR-21	Serum	↑ miR-21 in BC	↑ miR-21 in advanced BC	↑ miR-21 linked to ↑ radioresistance	↑ miR-21 in BC ↓ survival	NIA	[36]
miR-125b	Serum	NIA	↑ miR-125b linked to ↑ disease staging	↑ miR-125b linked to ↑ chemoresistance	NIA	NIA	[61]
miR-140-5p	Plasma	↓ miR-140-5p in BC as compared to CT	↓ miR-140-5p linked to worst disease prognosis	↓ miR-140-5p linked to ↑ chemoresistance	↓ miR-140-5p linked to ↓ EFS	↓ miR-140-5p linked to ↑ relapse/recurrence	[39]
miR-335	Serum	↓ miR-335 in BC as compared to CT	↓ miR-335 in TNBC	NIA	↓ miR-335 linked to ↓ OS	↓ miR-335 linked to ↑ relapse/recurrence	[54]
miR-34a/b/c	Plasma	↓ In the 3 miRNAs levels in BC as compared to CT	↓ miR-34a levels linked to advanced clinical staging & histopathological grading	NIA	↓ miR-34a levels linked to ↓ survival	NIA	[37]
miR-21, miR-23b, miR-200c, miR-190	Plasma	↑ miR-21, miR-23b & miR-200c levels & ↓ miR-190 in BC	The 4 miRNAs distinguished relapsed & non-relapsed BC cases	NIA	↑ miR-21 & miR-200c linked to short DFS	↑ miR-21, miR-23b & miR-200c & ↓ miR-190 in relapsed as compared to non-relapsed case	[42]
miR-16-5p, miR-17-3p, miR-451a, miR-940	Serum	No significant difference in the 4 miRNAs levels between BC & CT cases	The 4 miRNAs distinguished metastatic & non-metastatic BC cases	↓ In the 4 miRNAs in trastuzumab-resistant BC	↑ In the 4 miRNAs in improved BC survival	↑ In the 4 miRNAs in reduced incidence of relapse/recurrence	[50]
miR-18b, miR-103, miR-107, miR-652	Serum	↑ In the 4 miRNAs levels in TNBC	↑ In the 4 miRNAs levels linked to advanced clinical staging & histopathological grading	NIA	↑ In the 4 miRNAs ↓ RFS & OS	↑ In the 4 miRNAs in relapse group	[66]
Let-7a, miR-10b, miR-21, miR-145, miR-181a	Plasma	↑ miR-10b, miR-21 & miR-181a & ↓ let-7a & miR-145 in BC	↑ miR-10b, miR-21 & miR-181a & ↓ let-7a & miR-145 in locally advanced BC	NIA	↑ miR-10b & ↓ miR-21 linked to survival	↑ miR-10b & miR-21 linked to ↑ relapse	[57]
miR-26b-5p, miR-106b-5p, miR-142-3p, miR-142-5p, miR-185-5p, miR-362-5p	Whole blood	↑ In the 6 miRNAs levels in BC	↑ In the 6 miRNAs levels in early BC	NIA	↑ In the 6 miRNAs levels linked to ↓ OS/DFS	NIA	[45]
miR-19a, miR-19b-3p, miR-22-3p, miR-25-3p, miR-93-5p, miR-199a-3p, miR-210-3p	Plasma	↑ In the 7 miRNAs levels in BC	These miRNAs predicted BC patient survival & relapse	The 7 miRNAs regulate chemotherapy & targeted therapy resistance	↑ miR-19a, miR-19b, miR-93 & miR-201 linked to poor OS in TNBC patients	NIA	[30]
miR-296-3p, miR-575, miR-3610-5p, miR-4483, miR-4710, miR-4755-3p, miR-5698, miR-8089	Serum	↑ In the 8 miRNAs levels in BC	The 8 miRNAs distinguished metastatic & non-metastatic BC cases	NIA	↓ miR-5698 & miR-8089 linked to ↑ improved survival	The 8 miRNAs predicted distant metastases	[31]

BC: breast cancer; DCIS: ductal carcinoma in situ; DFS: disease-free survival; EFS: event-free survival; ER: estrogen receptor; LN: lymph node; OS: overall survival; NACT: neoadjuvant chemotherapy; TNBC: triple-negative breast cancer; PFS: progression-free survival; PR: progesterone receptor; NIA: no information available. The complete list of miRNA studies surveyed in this review is provided as Supplementary Tables S1 and S2.

**Table 4:** Potential exosomal and extracellular-vesicle miRNAs in diagnosing and predicting the prognosis or treatment responses among breast cancer patients. ↑ increase; ↓ reduce.

miRNAs	miRNAs Source	Diagnostic Significance	Significance in Grading/Classification	Prognostic Significance			Ref.
				Response to Treatment	Overall Survival	Relapse/Recurrence	
miR-24-3p	Plasma	↑ miR-24-3p in BC	↑ miR-24-3p linked to advanced clinical & histopathological grading	NIA	↓ miR-24-3p linked to improved survival	NIA	[47]
miR-363-5p	Plasma	↓ miR-363-5p in BC	↓ miR-363-5p in LN+ve BC cases as compared to LN –ve BC cases	NIA	↑ miR-363-5p linked to ↑ survival	NIA	[25]
miR-141, miR-200c	Plasma	↑ miR-141 & miR-200c in BC	↑ miR-141 in invasive BC; ↑ miR-141 & miR-200c in metastatic BC	NIA	↑ miR-200c linked to short OS	NIA	[64]
miR-155, miR-1246	Plasma	↑ Both miRNAs in trastuzumab-resistant BC	↑ Both miRNAs advanced BC as compared to non-advanced BC	↑ Both miRNAs in trastuzumab-resistant BC	↑ Both miRNAs linked to poor survival	↑ Both miRNAs linked to relapse & poor EFS	[38]
miR-21, miR-105, miR-222	Serum	↑ In the 3 miRNAs levels linked to presence of circulating BC cells	↑ miR-222 linked to advanced clinical staging & histopathological grading; ↑ miR-21 & miR-105 in metastatic than non-metastatic BC	↑ miR-21 reduced NACT response	NIA	NIA	[58]
miR-150-5p, miR-576-3p, miR-4665-5p	Plasma	↑ In the 3 miRNAs levels in BC	The 3 miRNAs distinguished recurrence & non-recurrence in BC cases	NIA	NIA	↑ In the 3 miRNAs levels in recurrent BC as compared to non-recurrent BC	[34]
miR-16, miR-30b, miR-93	Plasma	↑ miR-16 in BC & ↑ miR-93 in DCIS	↑ miR-93 in ER & PR +ve BC	NIA	NIA	↓ miR-30b linked to recurrence	[63]
miR-195-5p, miR-548ab, miR-2392, miR-2467-3p, miR-4448, miR-4800-3p	Serum	↑ miR-2392, miR-2467-3p, miR-4448 & miR-4800-3p levels in BC	The 6 miRNAs distinguished recurrence & non-recurrence in BC cases	↑ miR-2392, miR-2467-3p, miR-4448 & miR-4800-3p levels in BC with complete NACT response	↑ miR-2392, miR-2467-3p, miR-4448 & miR-4800-3p levels linked to ↑ OS in BC	↑ In miR-195-5p & ↓ miR-548ab in recurrent BC cases	[60]
miR-30b, miR-34a, miR-127, miR-141, miR-182, miR-183, miR-328, miR-423	Plasma	Dysregulation in the 8 miRNA levels in BC	↓ miR-30b, miR-127 & miR-328 in invasive BC	↑ miR-127 & miR-141 linked to complete NACT response; ↑ miR-34a, miR-182 & miR-183 linked to poor NACT response	↓ miR-141, miR-34a, miR-423, miR-182 & miR-183 linked to ↑ OS	NIA	[59]

BC: breast cancer; DCIS: ductal carcinoma in situ; DFS: disease-free survival; EFS: event-free survival; ER: estrogen receptor; LN: lymph node; OS: overall survival; NACT: neoadjuvant chemotherapy; TNBC: triple-negative breast cancer; PFS: progression-free survival; PR: progesterone receptor; NIA: no information available. The complete list of miRNA studies surveyed in this review is provided as Supplementary Tables S1 and S2.

### 3.3. Multifunctional Roles of Circulating miRNAs as Potential Biomarker for Human Breast Cancer

**Figure 1:**
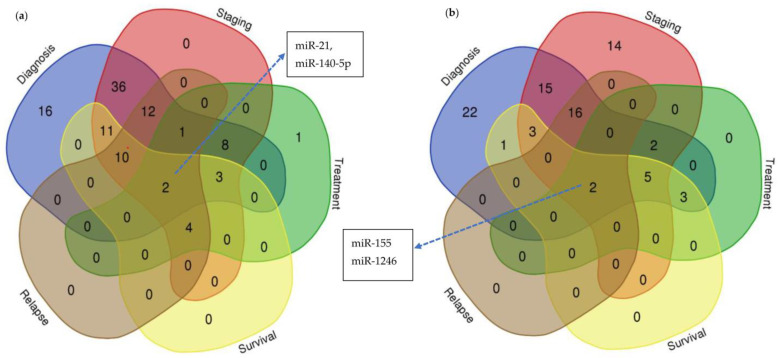
Number of (**a**) circulating and (**b**) exosomal and extracellular-vesicle (EV) miRNAs that play key roles in diagnosing and predicting prognosis or treatment responses among breast cancer patients. Number of (**a**) free-circulating (non exosomal) and (**b**) exosomal and extracellular-vesicle (EV) miRNAs. Numbers in the chart indicating the amount of miRNAs with the dedicated roles.

Evidently, circulating miRNAs have great potential to be employed as minimally invasive biomarkers in diagnosing BC at an early stage and complementary to the distinguishment of BC based on its clinical and histopathological grading [62,67]. In addition, circulating miRNAs are also helpful to predict the likelihood of relapse, recurrence and treatment responses among BC patients and this is particularly useful in guiding clinicians in planning a personalized treatment approach for different BC patients [30,68]. Given the challenge in identifying a miRNA panel useful for these functions within the growing number of related studies, we hope to provide an overview of the current status of the reported multifunctional roles of circulating miRNAs. Based on our findings from Table 3 and Table 4 as well as the Supplementary Tables S1 and S2, the miRNAs were further classified based on their reported roles in the diagnosis, staging classification and the prediction for relapse, treatment outcome and survival prognosis for BC patients (Figure 1a,b). We found that most of the reported free-circulating miRNAs were suitable in achieving the purpose of diagnosis only or concurrently in diagnosis and staging classification (Figure 1a). On the other hand, more reported exosomal and EV miRNAs were classified with either diagnosis only or staging only (Figure 1b), suggesting that exosomal and EV miRNAs may have more specific targets than free-circulating miRNA to be translated into clinical validation for diagnostic and staging purposes. Interestingly, there are two free-circulating (miR-21, miR-140-5p) and exosomal and EV (miR-155, miR-1246) miRNAs that were reported with the multifunctional roles, i.e., diagnosis, staging, treatment, survival and relapse.

### 3.4. Sensitivity and Specificity Levels of miRNA Detection in BC Patients

Several potential miRNA biomarkers have been identified in BC patients’ serum or plasma. With a receiver operating characteristic (ROC) curve analysis, miR-21-5p was shown to have greater potential in discriminating between BC patients and the control group than that of miR-221-3p [69]. Additionally, a recent meta-analysis on miR-21-5p and BC that comprises six publications, consisting of Asian and Caucasian study cohorts, further confirmed the potential early diagnostic role of miR-21-5p in BC patients due to its high pooled AUC and diagnostic odds ratio [70]. Further, another recent meta-analysis conducted with the aim of determining the overall diagnostic performance of 56 eligible studies involving circulating miRNAs via qPCR revealed a pooled sensitivity and specificity of 0.85 and 0.83, respectively [71]. Moreover, multiple miRNA panels with sensitivity and specificity scores of 0.90 and 0.86, respectively, were significantly higher compared to that of the single miRNA panels with corresponding sensitivity and specificity scores of 0.82 and 0.83, respectively. With regard to specimen type, pool sensitivity and specificity of plasma were 0.83 and 0.85, respectively, and the pool sensitivity and specificity of serum were 0.87 and 0.83, respectively, indicating little difference in the diagnostic performance between serum and plasma samples. These studies revealed that cell-free circuiting miRNA could function as a promising early diagnostic biomarker for the detection of BC [71].

## 4. Current Challenges and Issues in Circulating miRNAs as a Common Candidate for Liquid Biopsy in BC Management

While establishing significant dysregulated miRNA expression of significance blood samples, studies have shown contrary findings between the use of serum or plasma as starting materials for obtaining miRNAs and in multicentered heterogenous patient samples, as well as in the use of various statistical models. Nevertheless, the need for standardization of pre-analytical variables, namely sample processing, storage and handling, as well as the data normalization strategy for the quantification of miRNA, are often highlighted as possible causes for the discordant outcomes of the identified miRNA as diagnostic, predictive or prognostic markers [72].

### 4.1. Biological Parameters

The expression profiles between miRNAs obtained from human BC serum versus tumors using RNA-sequencing revealed a total of 109 significant differentially expressed miRNAs between the patient’s serum and healthy individuals’ serum. Furthermore, 174 significant differentially expressed miRNAs between normal tissues and tumors were observed, of which only 10 common miRNAs were differentially expressed in serum and tumor biopsy [73]. Furthermore, an in-depth analysis of data obtained from the HMDD v3.0 database and individual papers showed circulating miRNAs as BC diagnostic biomarkers lack specificity due to different expressions in tissues and blood of cancer patients and even miR-21-5p being cited as the most commonly dysregulated miRNA in BC studies was shown to be highly expressed in other cancer types and diseases [74]. On the other hand, a study on circulating miRNA among BC tumors, serum and normal tissues using microarray and qPCR was able to show that miRNA profiles between tumors matched to that of their corresponding serums, indicating the possible selective release of miRNAs from the tumor site to the blood [75]. In another study, in which the influence of the heterogeneous population setting on miRNA profile was obtained from two different geographical populations, one from Belgium (n = 110; primary BC = 55, healthy individuals = 55) and another from Rwanda (n = 110; primary BC = 55, healthy individuals = 55), using qPCR from plasma samples, revealed two distinct pools of circulating miRNA corresponding to each of the studied populations [76]. However, a multicenter study comprising Caucasian and Asian ethnicities from five different geographical locations obtained a common pool of miRNA, with AUC values ranging from 0.88 to 0.97 in the detection of early BC [15].

### 4.2. Statistical Models

The detection of dysregulated miRNA expression level methods requires normalization to remove variations across samples and different normalization methods were shown to have likely contributed to differences in the miRNA profiles obtained. In one study, qPCR was validated using the >2-fold change method from plasma circulating miRNAs and obtained 24 significant upregulated miRNAs and 16 significant downregulated miRNAs in BC patients compared to controls; however, only three miRNAs (miR-22-5p, miR-27b-3p, miR-423-5p) were able to distinguish cancer patients from healthy individuals [77]. Another study also used qPCR for the detection of possible circulating miRNA biomarkers in BC by creating a panel from an unbiased exploration among all expressed miRNAs via the two-fold cross-validation consolidating logistic regression and feature selection algorithm in the discovery cohort. Results from the study revealed the identification of six miRNA potential biomarker panels with an AUC of 0.78 and 0.77 in the discovery and validation cohorts, respectively, using the global geometric mean normalization method [15].

It is obvious that numerous studies (Table 3 and Table 4) have identified dysregulated miRNA profiles, which significantly represent early cancer detection, molecular subtype classification status and monitoring signatures of recurrence and metastatic BC progression. However, these studies often reveal a diverse pool of miRNA, with roles typically associated with the various hallmarks of cancer [78]. Therefore, by consolidating the big data of miRNA with artificial intelligence (AI) and machine learning platforms (ML), a more comprehensive and inclusive approach may be established for complementing clinical-based decisions in promoting effective BC patient care and recovery outcome.

## 5. Machine Learning and Deep Learning Approaches in BC Research

The field of healthcare has been transformed by technological advancements, such as the generation of large digital datasets. Over the past few decades, many researchers have put their efforts in exploring the application of machine learning (ML) in various healthcare applications, including cancer detection, diagnoses, prognoses, treatment and recurrence prediction [79,80,81,82]. Basically, there are two main common types of ML techniques, i.e., (i) supervised learning and (ii) unsupervised learning. The main difference between these two types of learning is the need for labelled training data. Supervised learning relies on labelled input and output training data. Based on the input and output data, the model will first identify their relationship before it can be used to classify new and unseen datasets and predict outcomes. On the contrary, unsupervised learning processes unlabeled or raw data. It is often used to identify the trends or patterns in raw datasets and perform initial data analysis.

### 5.1. Machine Learning and Deep Learning for Detection and Diagnosis

Detection and diagnosis of BC at the early stage helps in reducing the fatality rate to a greater extent. Rana et al. [83] conducted a comparative experiment with four different supervised algorithms, including the support vector machine (SVM), logistic regression (LR), k-nearest neighbor (KNN) and Naïve Bayes (NB) on the Wisconsin Breast Cancer Diagnostic dataset (WBCD) in predicting and diagnosing BC. Based on their analysis, the KNN technique provided the best results. NB and LR have also performed well in BC diagnosis. Nonetheless, they highlighted that SVM is a strong predictive and sophisticated machine learning algorithm, especially when it comes to predictive analysis; thus, this technique is also the most suited technique for recurrence or non-recurrence prediction of BC. Similar findings were also found by many researchers [84,85], whereby SVM has demonstrated its efficiency in BC prediction and diagnosis and achieved the best performance in terms of accuracy and precision.

In addition to BC detection and diagnosis, machine learning techniques were also used for subtypes classification. Three different ML techniques, including fuzzy SVM, Bayesian classifier and random forest (RF), were compared in categorizing the types of cancer from a sequence of mammography images in the MIAS database. They found that fuzzy SVM has the best performance compared to other ML techniques, with over 90% accuracy, sensitivity, specificity, precision and recall [86]. Similar findings were also obtained by Wu and Hicks [87], whereby the SVM technique was effective in discriminating the existence of triple-negative breast cancer (TNBC) based on the RNA sequencing datasets. The clinical significance of this investigation is that ML algorithms could be used not only to improve diagnostic accuracy, but also for identifying women who are at high risk of developing TNBC, which could be prioritized for treatment.

ML techniques have been widely utilized for cancer prognosis and survival prediction purposes too. For instance, in a study [88] involving eight different ML techniques to develop models for identifying and visualizing relevant prognostic indications of BC survival rates, based on 5 years’ BC patient database of the National Cancer Institute’s SEER Program from 2006 to 2010, the RF technique was found to be the best technique, with an accuracy level of 94.64%. A study focusing on the analyses of the impact of chemotherapy and establishment of prediction model of prognosis in early elderly TNBC was conducted by using machine learning, with 4696 patients in the SEER Database who were 70 years or older, diagnosed with primary early TNBC, from 2010 to 2016 [89]. The propensity-score-matched method was utilized to reduce covariable imbalance. Univariable and multivariable analyses were used to compare BC-specific survival (BCSS) and overall survival (OS). Nine models were developed by ML to predict the 5-year OS and BCSS for patients who received chemotherapy. The multivariate analyses showed a better survival in the chemotherapy group and the Light Gradient Boosting Machine (LightGBM) is a practical model for predicting survival and providing precious systemic treatment for patients who received chemotherapy [90].

Researchers have also used deep learning (DL), a subset of ML, in cancer applications. Based on the literature review, it was noted that some of the DL applications were found to have better performance compared to the conventional ML techniques. For instance, an ensemble deep learning approach for the definite classification of non-carcinoma and carcinoma BC histopathology images was able show a sensitivity of 97.73% for carcinoma classification, with an overall accuracy of 95.29% [91]. On the other hand, particle-swarm-optimized wavelet neural network (PSOWNN) was found relatively superior compared to other conventional ML techniques, such as CNN, KNN and SVM [92]. Meanwhile, the deep-learning-assisted efficient AdaBoost algorithm (DLA-EABA), a combined ML approach with AdaBoost algorithm as the base, for early BC detection showed a high accuracy level of 97.2%, sensitivity at 98.3% and specificity at 96.5% [93]. Apart from that, this method was reported to increase the patient survival rate.

### 5.2. Studies with miRNAs as Breast Cancer Biomarkers with ML/DL Approaches

MicroRNAs (miRNAs) have been suggested as the biomarkers or therapeutic targets in BCs [94,95,96]. However, there are not many BC studies utilizing the ML or DL approaches on miRNA biomarkers. Table 5 summarizes some of the related work for BC that adopted ML or DL approaches on miRNA biomarkers.

As the amount of miRNA expression data in the Genomic Data Commons (GDC) Data Portal increased dramatically, several researchers proposed feature-selection methods to reduce the size of datasets, before they proceed with their analysis. An ensemble feature-selection methodology for miRNA signatures was proposed to identify the most robust and reliable miRNAs to be used in clinically relevant prediction tasks [97]. In their research, including over 8000 samples from TCGA, 100 miRNA signatures were identified and distinguished between tumor and normal tissues. The proposed approach provided better accuracy after 10-fold cross-validation with different ML classifiers, showing over 90% classification accuracy. In another study, the ensemble methodology was performed to identify the important biomarkers for BC and then classified by different ML techniques, such as NB, LR, KNN, SVM and multilayer perceptron [98]. In their preliminary analysis, default parameters were changed only when experimentation showed that classifier performance generally improved significantly across all datasets. Rehman et al. [99] performed four different feature-selection methods, including the Information Gain (IG), Chi-Squared (CHI2) and Least Absolute Shrinkage and Selection Operation (LASSO), to identify the most specific and effective miRNAs in discriminating normal and cancerous tissues. After feature selection, they applied the RF and SVM algorithms to identify the cancerous cell. The study demonstrated that the miRNAs ranked higher by their analysis had higher classifier performance. Performance becomes lower as the rank of the miRNA decreases, which shows that these miRNAs had different degrees of importance as biomarkers.

The tree-based ML models were normally applied on specific miRNAs for classifying the upregulated and downregulated BC cells [100]. In addition, several supervised methods, such as DT, NB, neural network and DL, were adopted, to classify cancer cells based on the expression of the microRNA gene to obtain the best method that can be used for gene analysis [101]. It was found that the DL method, which was developed based on a multilayer feed-forward ANN trained with stochastic gradient descent using backpropagation outperformed other conventional ML methods [101].

## 6. Conclusions

Key cancer signatures are represented by the dysregulated expression of miRNAs detected in liquid biopsy and shown to be associated with BC diagnosis, subtype classification and recurrence, as well as metastatic spread of the cancerous cells. Therefore, using miRNA-based liquid biopsy may be much more feasible in BC management, which also may come with greater patient acceptance as it could be conducted as simple routine blood collection, but the amount of information contained in the miRNA profile is immense. Using bioinformatics and with current and emerging AI and ML platforms, this huge amount of miRNA data may be able to be analyzed in ways that provide cancer progression indicators that complement standard clinical practices. However, translation of the miRNA targets selected by ML requires clinical validation to achieve the number of biomarkers that can accurately perform the expected roles cost effectively. This will eventually result in better treatment outcomes and supportive BC management from early detection to personalized therapy, ultimately improving the quality of life among BC patients.

## Figures and Tables

**Table 1:** Clinical and molecular classification of BC.

Cancer Type	Benign	Pre-Malignant/ In-Situ (20–25%) [13]	Malignant/Invasive [IDC (80%), ILC (20%)] [13]					
Categories	Fibroadenoma Intraductal papilloma Lipoma	Early Breast Cancer Detection	Molecular Subtypes (St Gallen)					Recurrence/ Metastatic
	Lubular Carcinoma In-situ (LCIS)	Ductal Carcinoma In-situ (DCIS)	Luminal A	Luminal B (HER2-)	Luminal B (HER2+)	HER2+ Enriched	TNBC	
Cancer/Bio markers [11,12]		ER, PR, HER2 & Ki67 (low < 10%); Germline test BRCA1 & 2 (High risk group)	ER+; PR+; HER2−; Ki67 low (<10–14%); Germline test BRCA1 & 2 (High Risk Group)	ER+; PR−; HER2−; Ki67 high (>14–30%); Germline test BRCA1 & 2 (High Risk Group)	ER+; PR+/−; HER2+; Ki67 high/low; Germline test BRCA1 & 2 (High Risk Group)	ER−; PR−; HER2+; Ki67 high; Germline test BRCA1 & 2 (High Risk Group)	ER−; PR−; HER2−; Ki67 high; (CK 5/6+; EGFR+); Germline test BRCA1 & BRCA2 (High Risk Group)	Metastatic Site: Bone, liver, lungs, brain ESCAT score: I = Good prognosis II = Poor Prognosis
Frequency of cases [14]		20–25%	40–50%	20–30%	20–30%	15–20%	10–20%	4%
Histological grade (Majority)			Well differentiated (G1)	Moderately differentiated (G2)	Moderately differentiated (G2)	Poorly differentiated (G3)	Poorly differentiated (G3)	Poorly differentiated (G4)
TNM Stage		NR	I-III	I-III	I-III	I-III	I-III	IV
Prognosis		NR	Good	Intermediate	Intermediate	Poor	Poor	Poor
Response to therapies [11,12,14]		Surgery Breast-conserving surgery (BCS) Radiotherapy Lumpectomy Mastectomy	Endocrine	Endocrine Chemotherapy	Endocrine Chemotherapy Targeted Therapy	Chemotherapy Targeted Therapy	Chemotherapy PARP inhibitors	Chemotherapy CKD4/6 Inhibitor Fulvestrant

Abbreviations: BRCA 1 & 2: Breast Cancer gene 1 and 2; TNM: tumor, node, and metastasis; DCIS: Ductal Carcinoma In-situ; ESCAT: ESMO Scale for Clinical Actionability of molecular Targets; ER: Estrogen receptor; HER2: Human epidermal growth factor receptor; IDC: Invasive Ductal Carcinoma; ILC: Invasive Lobular Carcinoma; LCIS: Lubular Carcinoma In-situ; PALB2: Partner and localizer of BRCA2; PDL1: Program death-ligand 1; PR: Progesterone receptor, 2; MSI: Microsatellite instability.

**Table 2:** Clinical trials that involved circulating miRNAs as liquid biopsy of human breast cancer (n = 2).

Study Name	Year Launched	Study ID	Location	Status
Onco-liq: Kit for Breast Cancer Diagnosis.	2021	NCT04906330	Argentina	On-going
Prospective Breast Cancer Biobanking (PBCB)	2020	NCT04488614	Norway	On-going

**Table 5:** Related work for BC studies with miRNA as biomarkers.

Reference	Function/Purpose	Methods	Accuracy of Model
[97]	Cancer Classification	Gradient Boosting	Accuracy 93.59%
		RF	Accuracy 93.24%
		LR	Accuracy 92.37%
		Passive Aggressive	Accuracy 88.31%
		SGD	Accuracy 90.35%
		SVM	Accuracy 91.54%
		Ridge	Accuracy 83.05%
		Bagging	Accuracy 91.1%
[98]	Cancer Classification	NB	Accuracy 94.9%
[99]	Cancer detection	RF	AUC 99.5–99.9%
		SVM	AUC 93.8–99.6%
		ANN	Accuracy 97.3%
		KNN	Accuracy 99.2%
		SVM	Accuracy 96.3%
		LR	Accuracy 95.8%
[100]	Cancer Classification	Tree-based model	NIA
[101]	Cancer Classification	DT	Accuracy 99.12%
		NB	Accuracy 93.86%
		ANN	Accuracy 100%
		DL	Accuracy 100%

Abbreviations: BC: breast cancer; SVM: Support Vector Machine; LR: Logistic Regression; KNN: k-Nearest Neighbor; NB: Naïve Bayes; WBCD: Wisconsin Breast Cancer Diagnostic dataset; RF: Random Forest; SGD: Stochastic Gradient Descent; ANN: Artificial Neural Network; DT: Decision Tree; DL: Deep Learning; NIA: no information available.

## Data Availability

No new data were created or analyzed in this study. Data sharing is not applicable for this study.

## Supplementary Materials

The supporting information can be downloaded at: https://www.mdpi.com/article/10.3390/ijms232315382/s1.
