# Supplementary material for: Addressing the Clinical Feasibility of Adopting Circulating miRNA for Breast Cancer Detection, Monitoring and Management with Artificial Intelligence and Machine Learning Platforms
Source: Int J Mol Sci. 2022 Dec 6;23(23):15382. doi: 10.3390/ijms232315382 (PMC9736108; doi:10.3390/ijms232315382)
Supplement: Supplementary file 1 [file ijms-23-15382-s001.zip › ijms-2029353-supplementary.pdf]

Review

Addressing the Clinical Feasibility of Adopting Circulating miRNA for Breast Cancer Detection, Monitoring and Management with Artificial Intelligence and Machine Learning Platforms

Lloyd Ling [1], Ahmed Faris Aldoghachi [2], Zhi Xiong Chong [3], Wan Yong Ho [3], Swee Keong Yeap [4], Ren Jie Chin [1], Eugene Zhen Xiang Soo [1], Jen Feng Khor [1], Yoke Leng Yong [5], Joan Lucille Ling [6], Naing Soe Yan [2] and Alan Han Kiat Ong [2,*]

[1] Lee Kong Chian Faculty of Engineering & Science, Universiti Tunku Abdul Rahman, Jalan Sungai Long, Bandar Sungai Long, 43000 Kajang, Malaysia

[2] M. Kandiah Faculty of Medicine and Health Sciences, Universiti Tunku Abdul Rahman, Cheras 43000, Malaysia

[3] Division of Biomedical Sciences, School of Pharmacy, Faculty of Sciences and Engineering, University of Nottingham Malaysia, 43500 Semenyih, Malaysia

[4] China-ASEAN College of Marine Sciences, Xiamen University Malaysia, 43900 Sepang, Selangor, Malaysia

[5] Department of Computing and Information Systems, Sunway University, No. 5, Jalan Universiti, Bandar Sunway, 47500 Petaling Jaya, Selangor, Malaysia

[6] Arizona State University, Edson College of Nursing and Health Innovation, Phoenix, AZ 85004, USA

* Correspondence: onghk@utar.edu.my

Table S1. Roles of free-circulating miRNAs in diagnosing and predicting the prognosis or treatment responses among breast cancer patients.

miRNAs	Study design (miRNAs source)	Detection/ validation method (RNA-seq/qPCR)	Diagnostic significance	Significance in grading/classification	Prognostic significance			Ref
					Response to treatment	Overall survival	Relapse/Recurrence	
miR-21	Serum (10 BC)	qPCR	↑ miR-21 in BC as compared to CT	↑ miR-21 in advanced BC	↑ miR-21 linked to ↑ radioresistance	↑ miR-21 in BC ↓ survival	NIA	[1]
miR-21	Plasma (26 BC, 16 CT)	qPCR	↑ miR-21 in BC as compared to CT	↑ miR-21 in advanced BC	NIA	NIA	NIA	[2]
miR-106a	Plasma/serum (50 BC, 30 CT)	qPCR	↑ miR-106a in BC as compared to CT	↑ miR-106a in LN +ve BC as compared to LN –ve BC	NIA	NIA	NIA	[3]
miR-125b	Serum (56 BC)	qPCR	NIA	↑ miR-125b linked to ↑ disease staging	↑ miR-125b linked to ↑ chemoresistance	NIA	NIA	[4]
miR-140-5p	Plasma (455 BC)	qPCR	↓ miR-140-5p in BC as compared to CT	↓ miR-140-5p linked to worst disease prognosis	↓ miR-140-5p linked to ↑ chemoresistance	↓ miR-140-5p linked to EFS ↓	↓ miR-140-5p linked to ↑ relapse/recurrence	[5]
miR-182	Serum (46 BC, 58 CT)	qPCR	↑ miR-182 in BC as compared to CT	↓ miR-182 in both ER & PR +ve BC	NIA	NIA	NIA	[6]
miR-200c	Serum (46 BC)	qPCR	NIA	↑ miR-200c in TNBC as compared ER & PR +ve BC	NIA	NIA	NIA	[7]
miR-335	Serum (106 BC, 40 CT)	qPCR	↓ miR-335 in BC as compared to CT	↓ miR-335 in TNBC	NIA	↓ miR-335 linked to ↓ OS	↓ miR-335 linked to ↑ relapse/recurrence	[8]
miR-373	Serum (196 BC, 125 CT)	qPCR	↑ miR-373 in BC as compared to CT	↑ miR-373 linked to advanced clinical staging & histopathological grading	NIA	NIA	NIA	[9]

miR-484	Serum (98 BC, 25 CT)	LDA & qPCR	↑ miR-484 in BC as compared to CT	↑ miR-484 in early BC	NIA	NIA	NIA	[10]
miR-21<br>miR-155	Serum (40 BC, 20 benign cases)	qPCR	↑ Both miRNAs levels in BC as compared to benign cases	NIA	NIA	NIA	NIA	[11]
miR-19a<br>miR-205	Serum (68 BC)	miRNA microarray & qPCR	NIA	NIA	↑ Both miRNAs in treatment-resistant BC	NIA	NIA	[12]
miR-152<br>miR-210	Plasma (30 BC, 20 CT)	qPCR	↑ miR-210 & ↓ miR-152 in BC as compared to CT	↑ miR-210 linked to advanced clinical staging	NIA	NIA	NIA	[13]
miR-133a<br>miR-148b	Plasma (127 BC, 85 CT)	qPCR	↑ Both miRNAs levels in BC as compared to CT	↑ Both miRNAs levels in early stage BC as compared to CT	NIA	NIA	NIA	[14]
miR-17<br>miR-25<br>miR-133	Serum (100 BC, 142 CT)	qPCR	↑ miR-17 & ↓ miR-25 & miR-133 in BC as compared to CT	↑ miR-17 & ↓ miR-25 & miR-133 linked to advanced clinical staging	NIA	↑ miR-17 linked to ↓ survival	NIA	[15]
miR-34a<br>miR-34b<br>miR-34c	Plasma (173 BC, 75 CT)	qPCR	↓ In the 3 miRNAs levels in BC as compared to CT	↓ miR-34a levels linked to advanced clinical staging & histopathological grading	NIA	↓ miR-34a levels linked to ↓ survival	NIA	[16]
Let-7a<br>miR-10b<br>miR-21<br>miR-195	Plasma/serum (148 BC, 44 CT)	qPCR	↑ miR-195 in BC as compared to CT	↑ miR-10b & miR-21 in ER –ve BC	NIA	NIA	NIA	[17]

miR-181a<br>miR-195<br>miR-329<br>miR-331	Plasma (4 BC, 4 CT)	miRNA-seq	↑ In the 4 miRNAs levels in BC as compared to CT	↓ miR-195 in metastatic BC as compared to CT	NIA	NIA	NIA	[18]
miR-21<br>miR-23b<br>miR-200b<br>miR-200c	Plasma (243 BC, 23 CT)	qPCR	↑ In the 4 miRNAs levels in BC as compared to CT	The 4 miRNAs distinguished metastatic & early BC cases	NIA	↑ miR-23b & miR-190 level linked to short PFS;<br>↑ miR-200b level linked to short OS	NIA	[19]
miR-21<br>miR-23b<br>miR-200c<br>miR-190	Plasma (209 BC, 23 CT)	qPCR	↑ miR-21, miR-23b & miR-200c levels & ↓ miR-190 in BC as compared to CT	The 4 miRNAs distinguished relapsed & non-relapsed BC cases	NIA	↑ miR-21 & miR-200c linked to short DFS	↑ miR-21, miR-23b & miR-200c & ↓ miR-190 in relapsed as compared to non-relapsed case	[20]
miR-16-5p<br>miR-17-3p<br>miR-451a<br>miR-940	Serum (386 BC, 55 CT)	miRNA microarray & qPCR	No significant difference in the 4 miRNAs levels between BC & CT cases	The 4 miRNAs distinguished metastatic & non-metastatic BC cases	↓ In the 4 miRNAs in trastuzumab-resistant BC	↑ In the 4 miRNAs in improved BC survival	↑ In the 4 miRNAs in reduced incidence of relapse/recurrence	[21]
miR-18b<br>miR-103<br>miR-107<br>miR-652	Serum (110 BC, 30 CT)	qPCR	↑ In the 4 miRNAs levels in TNBC as compared to CT	↑ In the 4 miRNAs levels linked to advanced clinical staging & histopathological grading	NIA	↑ In the 4 miRNAs ↓ RFS & OS	↑ In the 4 miRNAs in relapse group	[22]
miR-17<br>miR-34a<br>miR-93<br>miR-155<br>miR-373	Serum (152 BC, 40 CT)	qPCR	↑ miR-34a, miR-93 & miR-373 in BC as compared to CT	↓ miR-17 & miR-155 in metastatic BC as compared to non-metastatic BC<br>↓ miR-17 in ER & PR +ve BC<br>↑ miR-373 in HER2 +ve BC	NIA	NIA	NIA	[23]

Let-7b-5p<br>miR-122-5p<br>miR-146b-5p<br>miR-210-3p<br>miR-215-5p	Plasma (257 BC, 257 CT)	qPCR	↑ In the 5 miRNAs levels in BC as compared to CT	No significance association between the 5 miRNAs & clinico-pathological staging	NIA	NIA	NIA	[24]
Let-7a<br>miR-10b<br>miR-21<br>miR-145<br>miR-181a	Plasma (30 BC, 20 CT)	qPCR	↑ miR-10b, miR-21 & miR-181a & ↓ let-7a & miR-145 in BC as compared to CT	↑ miR-10b, miR-21 & miR-181a & ↓ let-7a & miR-145 in locally advanced BC	NIA	↑ miR-10b & miR-21 linked to ↓ survival	↑ miR-10b & miR-21 linked to ↑ relapse	[25]
miR-1307-3p<br>miR-1246<br>miR-4634<br>miR-6861-5p<br>miR-6875-5p	Serum (1280 BC, 2836 CT)	miRNA microarray & qPCR	↑ In the 5 miRNAs levels in BC as compared to CT	NIA	NIA	NIA	NIA	[26]
miR-21-5p<br>miR-96-5p<br>miR-125b-5p<br>miR-505-5p<br>miR-3656	Plasma (83 BC, 26 CT)	miRNA microarray & qPCR	Dysregulation in the 11 miRNA levels in BC as compared to CT cases	These 5 miRNAs predicted early BC occurrence	↑ miR-21-5p, miR-96-5p, miR-125b-5p & miR-505-5p in BC patients ↓ before treatment; miR-21-5p, miR-505-5p & miR-3656 in treated BC patients	NIA	NIA	[27]
miR-19b-3p<br>miR-20b-5p<br>miR-92a-3p<br>miR-92a-2-5p<br>miR-106a-3p<br>miR-106a-5p	Plasma (200 BC, 200 CT); serum (204 BC, 202 CT)	qPCR	↑ In the 6 miRNAs levels in BC as compared to CT	↑ miR-106a-5p & miR-20b-5p in HER2 -ve BC<br><br>↑ miR-106a-5p, miR-92a-2-5p & miR-20b-5p in ER +ve BC	NIA	NIA	NIA	[28]

miR-26b-5p<br>miR-106b-5p<br>miR-142-3p<br>miR-142-5p<br>miR-185-5p<br>miR-362-5p	Whole blood (74 BC, 19 CT)	miRNA microarray & qPCR	↑ In the 6 miRNAs levels in BC as compared to CT	↑ In the 6 miRNAs levels in early BC	NIA	↑ In the 6 miRNAs levels linked to ↓ OS/DFS	NIA	[29]
miR-21-5p<br>miR-194-5p<br>miR-205-5p<br>miR-375<br>miR-376c-3p<br>miR-382-5p<br>miR-411-5p	Serum (48 BC, 31 CT)	miRNA PCR panel and qPCR	Dysregulation in the 7 miRNAs levels in BC as compared to CT cases	Dysregulation in the 7 miRNAs levels linked to worst clinical prognosis	NIA	NIA	↑ miR-21-5p, miR-194-5p, miR-205-5p & miR-375 ↓ & miR-376c-3p, miR-382-5p & miR-411-5p in recurrent BC	[30]
miR-19a<br>miR-19b-3p<br>miR-22-3p<br>miR-25-3p<br>miR-93-5p<br>miR-199a-3p<br>miR-210-3p	Plasma (93 BC, 34 CT)	miRNA microarray & qPCR	↑ In the 7 miRNAs levels in BC as compared to CT	These miRNAs predicted BC patient survival & relapse	The 7 miRNAs regulate chemotherapy & targeted therapy resistance	↑ miR-19a, miR-19b, miR-93 & miR-201 linked to poor OS in TNBC patients	NIA	[31]
miR-127-3p<br>miR-148b<br>miR-376a<br>miR-376c<br>miR-409-3p<br>miR-652<br>miR-801	Plasma (247 BC, 140 CT)	miRNA microarray & qPCR	↑ miR-127-3p, miR-376a, miR-376c & miR-409-3p in BC as compared to CT	↑ miR-127-3p, miR-148b, miR-409-3p, miR-652 & miR-801 in early BC	NIA	NIA	NIA	[32]

miR-296-3p<br>miR-575<br>miR-3610-5p<br>miR-4483<br>miR-4710<br>miR-4755-3p<br>miR-5698<br>miR-8089	Serum (147 BC)	miRNA microarray	↑ In the 8 miRNAs levels in BC as compared to CT	The 8 miRNAs distinguished metastatic & non-metastatic BC cases	NIA	↓ miR-5698 & ↑ miR-8089 linked to improved survival	The 8 miRNAs predicted distant metastases	[33]
miR-21<br>miR-24<br>miR-202<br>miR-206<br>miR-219B<br>miR-223<br>miR-373<br>miR-1246<br>miR-6875	Plasma (146 CT, 226 BC)	qPCR	↑ In the 9 miRNAs levels in BC compared to CT	NIA	NIA	NIA	NIA	[34]
miR-188-5p<br>miR-642b-3p<br>miR-1202<br>miR-1207-5p<br>miR-1225-5p<br>miR-1290<br>miR-3141<br>miR-4270<br>miR-4281	Plasma/serum (23 BC, 9 CT)	miRNA microarray & qPCR	↑ In the 9 miRNAs levels in BC compared to CT	↑ In the 9 miRNAs levels in early BC (stage I, II & III) compared to late BC (stage IV)<br><br>↑ In the 9 miRNAs levels in TNBC & HER2 +ve BC than luminal BC	NIA	NIA	NIA	[35]

Table S2. Roles of exosomal and extracellular-vesicle (EV) miRNAs in diagnosing and predicting the prognosis or treatment responses among breast cancer patients.

miRNAs	Stud design (miRNAs source)	Detection/ validation method (RNA-seq/qPCR)	Diagnostic significance	Significance in grading/classification	Prognostic significance			Ref
					Response to treatment	Overall survival	Relapse/Recurrence	
miR-24-3p	Plasma (1780 BC)	Nanostring & miRNA-seq	↑ miR-24-3p in BC as compared to CT	↑ miR-24-3p linked to advanced clinical & histopathological grading	NIA	↓ miR-24-3p linked to ↑ improved survival	NIA	[36]
miR-122-5p	Plasma (32 BC, 32 CT)	qPCR	↑ miR-122-5p in BC as compared to CT	NIA	NIA	NIA	NIA	[24]
miR-223-3p	Plasma (3 IDC, 3 DCIS, 3 CT)	miRNA microarray & qPCR	↑ miR-223-3p in IDC as compared to DCIS & CT	↑ miR-223-3p linked to advanced clinical & histopathological grading	NIA	NIA	NIA	[37]
miR-363-5p	Plasma (10 BC, 10 CT)	miRNA-seq & qPCR	↓ miR-363-5p in BC as compared to CT	↓ miR-363-5p in LN+ve BC cases as compared to LN –ve BC cases	NIA	↑ miR-363-5p linked to ↑ survival	NIA	[38]
miR-451a	Serum (44 BC, 67 CT)	qPCR	↑ miR-451a in BC as compared to CT	No significant association between miR-451a level and BC severity	NIA	NIA	NIA	[39]
miR-4488	Serum (96 BC)	qPCR	↓ miR-4488 in BC as compared to CT	NIA	NIA	NIA	NIA	[40]
miR-141<br>miR-200c	Plasma (33 BC, 6 DICS, 51 CT)	qPCR	↑ miR-141 & miR-200c in BC as compared to control	↑ miR-141 in invasive BC; ↑ miR-141 & miR-200c in metastatic BC	NIA	↑ miR-200c linked to short OS	NIA	[41]
miR-21<br>miR-1246	Plasma (16 BC, 16 CT)	miRNA-seq & qPCR	↑ Both miRNAs in BC as compared to CT	↑ miR-21 & miR-1246 related to ↑ clinical staging & histopathological grading	NIA	NIA	NIA	[42]

miR-155<br>miR-1246	Plasma (8 BC)	miRNA microarray & qPCR	↑ Both miRNAs in trastuzumab-resistant BC	↑ Both miRNAs advanced BC as compared to non-advanced BC	↑ Both miRNAs in trastuzumab-resistant BC	↑ Both miRNAs linked to poor survival	↑ Both miRNAs linked to relapse & poor EFS	[43]
miR-21<br>miR-105<br>miR-222	Serum (53 BC, 8 CT)	qPCR	↑ In the 3 miRNAs levels linked to presence of circulating BC cells	↑ miR-222 linked to advanced clinical staging & histopathological grading<br><br>↑ miR-21 & miR-105 in metastatic than non-metastatic BC	↑ miR-21 reduced NACT response	NIA	NIA	[44]
miR-150-5p<br>miR-576-3p<br>miR-4665-5p	Plasma (27 BC, 3 CT)	miRNA-seq	↑ In the 3 miRNAs levels in BC as compared to CT	The 3 miRNAs distinguished recurrence & non-recurrence in BC cases	NIA	NIA	↑ In the 3 miRNAs levels in recurrent BC as compared to non-recurrent BC	[45]
miR-21-5p<br>miR-23a-3p<br>tRF-Lys	Serum (78 BC, 72 CT)	miRNA-seq	↑ In the 3 miRNAs levels in BC as compared to CT	↑ miR-21-5p, miR-23a-3p & tRF-Lys discriminated early stage BC from CT	NIA	NIA	NIA	[46]
miR-181b-5p<br>miR-222-3p<br>let-7a-5p	Plasma (23 IBC, 34 non-IBC, 20 CT)	qPCR	↑ miR181b-5p & miR-222-3p & ↓ let-7a-5p in IBC as compared to non-IBC	↑ miR-181b-5p & miR-222-3p ↓ & let-7a-5p in advanced IBC	NIA	NIA	NIA	[47]
miR-18a-3p<br>miR-136-5p<br>miR-4685-3p	Serum (17 BC, 16 CT)	miRNA-seq & qPCR	↑ In the 4 miRNAs levels in BC as compared to CT	NIA	NIA	NIA	NIA	[48]
miR-16<br>miR-30b<br>niR-93	Plasma (32 BC, 8 DCIS, 8 CT)	miRNA microarray & qPCR	↑ miR-16 in BC & ↑ miR-93 in DCIS	↑ miR-93 in ER & PR +ve BC	NIA	NIA	↓ miR-30b linked to recurrence	[49]

miR-142-5p<br>miR-150-5p<br>miR-320a<br>miR-4433b-5p	Serum (16 LA, 15 TNBC, 16 CT)	miRNA-seq & qPCR	↑ In the 4 miRNAs levels in BC as compared to CT	↓ miR-142-5p & miR-150-5p linked to ↑ tumour grades<br><br>↓ miR-142-5p & miR-320a linked to ↑ tumour sizes<br><br>miR-142-5p in LA>CT & TNBC<br><br>miR-150-5p in LA>TNBC<br><br>miR-320a & miR-4433-5p in LA>CT	NIA	NIA	NIA	[50]
miR-19b-3p<br>miR-92a-3p<br>miR-92a-2-5p<br>miR-106a-3p<br>miR-106a-5p	Plasma (32 BC, 32 CT); serum (32 BC, 32 CT)	qPCR	↑ In the 5 miRNAs levels in BC as compared to CT	NIA	NIA	NIA	NIA	[28]
miR-195-5p<br>miR-548ab<br>miR-2392<br>miR-2467-3p<br>miR-4448<br>miR-4800-3p	Serum (24 BC)	miRNA microarray & qPCR	↑ miR-2392, miR-2467-3p, miR-4448 & miR-4800-3p levels in BC as compared to CT	The 6 miRNAs distinguished recurrence & non-recurrence in BC cases	↑ miR-2392, miR-2467-3p, miR-4448 & miR-4800-3p levels in BC with complete NACT response	↑ miR-2392, miR-2467-3p, miR-4448 & miR-4800-3p levels linked to ↑ OS in BC	↑ In miR-195-5p & ↓ miR-548ab in recurrent BC cases	[51]
miR-30b<br>miR-34a<br>miR-127<br>miR-141<br>miR-182<br>miR-183<br>miR-328<br>miR-423	Plasma (20 BC)	miRNA-seq	Dysregulation in the 8 miRNA levels in BC as compared to CT cases	↓ miR-30b, miR-127 & miR-328 in invasive BC as compared to CT	↑ miR-127 & miR-141 linked to complete NACT response;<br>↑ miR-34a, miR-182 & miR-183 linked to poor NACT response	↓ miR-141, miR-34a, miR-423, miR-182 & miR-183 linked to ↑ OS	NIA	[52]

miR-338-3p<br>miR-340-5p<br>miR-124-3p<br>miR-17-5p<br>miR-18a-5p<br>miR-20b-5p<br>miR-29b-3p<br>miR-93-5p<br>miR-130-3p<br>miR-195-5p<br>miR-486-5p	Serum (32 BC)	miRNA PCR array & qPCR	Dysregulation in the 11 miRNA levels in BC as compared to CT cases	The 11 miRNAs distinguished BC recurrence from non-recurrence cases	NIA	NIA	↑ miR-338-3p, miR-340-5p, miR-124-3p & ↓ miR-17-5p, miR-18a-5p, miR-20b-5p, miR-29b-3p, miR-93-5p, miR-130-3p, miR-195-5p, miR-486-5p in recurrent BC as compared to non-recurrent cases	[53]
Let-7g<br>miR-16<br>miR-27a<br>miR-27b<br>miR-30c<br>miR-143<br>miR-148a<br>miR-150<br>miR-152<br>miR-199a-3p<br>miR-202<br>miR-335<br>miR-340<br>miR-365<br>miR-370<br>miR-376a<br>miR-376c<br>miR-382<br>miR-410<br>miR-422a<br>miR-433<br>miR-489<br>miR-598	Plasma (435 BC, 20 CT)	microRNA array	↑ miR-27a, miR-27b, miR-152, miR-199a-3p, miR-340, miR-376a, miR-410 & miR-598 & ↓ miR-30c, miR-150 in BC as compared to CT	↑ Let-7g, miR-148a, miR-202, miR-335, miR-370, miR-376c, miR-382, miR-422a, miR-433, miR-489, miR-628, miR-652 & miR-891a in TNBC as compared to HER2 +ve BC; ↓ miR-16, miR-27a, miR-27b, miR-143 & miR-365 in TNBC as compared to HER2 +ve BC	NIA	NIA	NIA	[54]

miR-628						
miR-652						
miR-891a						

BC: breast cancer; CT: healthy control; DCIS: ductal carcinoma *in situ*; DFS: disease-free survival; EFS: event-free survival; ER: estrogen receptor; HER2: human epidermal growth factor receptor 2; IBC: inflammatory breast cancer, IDC: invasive ductal carcinoma; LA: luminal A; LN: lymph node; OS: overall survival; LDA: low density array; NACT: neoadjuvant chemotherapy; TNBC: triple-negative breast cancer; miRNA-seq: microRNA-sequencing; PFS: progression-free survival; PR: progesterone receptor; qPCR: real-time polymerase chain reaction; NIA: no information available

References

1. Dan, T.; Shastri, A.A.; Palagani, A.; Buraschi, S.; Neill, T.; Savage, J.E.; Kapoor, A.; Deangelis, T.; Addya, S.; Camphausen, K.; et al. MiR-21 Plays a Dual Role in Tumor Formation and Cytotoxic Response in Breast Tumors. *Cancers (Basel)* **2021**, *13*, 888, doi:10.3390/cancers13040888.
2. Diansyah, M.N.; Prayogo, A.A.; Sedana, M.P.; Savitri, M.; Romadhon, P.Z.; Amrita, P.N.A.; Wijaya, A.Y.; Hendrata, W.M.; Bintoro, U.Y. Early Detection Breast Cancer: Role of Circulating Plasma MiRNA-21 Expression as a Potential Screening Biomarker. *Turk J Med Sci* **2021**, *51*, 562–569, doi:10.3906/sag-2005-138.
3. Mohmmed, E.A.; Shousha, W.G.; EL-Saiid, A.S.; Ramadan, S.S. A Clinical Evaluation of Circulating MiR-106a and Raf-1 as Breast Cancer Diagnostic and Prognostic Markers. *Asian Pacific Journal of Cancer Prevention* **2021**, *22*, 3513–3520, doi:10.31557/APJCP.2021.22.11.3513.
4. Wang, H.; Tan, G.; Dong, L.; Cheng, L.; Li, K.; Wang, Z.; Luo, H. Circulating Mir-125b as a Marker Predicting Chemoresistance in Breast Cancer. *PLoS One* **2012**, *7*, e34210, doi:10.1371/journal.pone.0034210.
5. Di Cosimo, S.; Appierto, V.; Pizzamiglio, S.; Tiberio, P.; Iorio, M. V.; Hilbers, F.; De Azambuja, E.; De La Peña, L.; Izquierdo, M.; Huober, J.; et al. Plasma MiRNA Levels for Predicting Therapeutic Response to Neoadjuvant Treatment in HER2-Positive Breast Cancer: Results from the NeoALTTO Trial. *Clinical Cancer Research* **2019**, *25*, 3887–3895, doi:10.1158/1078-0432.CCR-18-2507.
6. Wang, P.-Y.; Gong, H.-T.; Li, B.-F.; Lv, C.-L.; Wang, H.-T.; Zhou, H.-H.; Li, X.-X.; Xie, S.-Y.; Jiang, B.-F. Higher Expression of Circulating MiR-182 as a Novel Biomarker for Breast Cancer. *Oncol Lett* **2013**, *6*, 1681–1686, doi:10.3892/ol.2013.1593.
7. Niedźwiecki, S.; Piekarski, J.; Szymańska, B.; Pawłowska, Z.; Jeziorski, A. Serum Levels of Circulating MiRNA-21, MiRNA-10b and MiRNA-200c in Triple-Negative Breast Cancer Patients. *Ginekol Pol* **2018**, *89*, 414–419, doi:10.5603/GP.a2018.0071.
8. Swellam, M.; Mahmoud, M.S.; Hashim, M.; Hassan, N.M.; Sobeih, M.E.; Nageeb, A.M. Clinical Aspects of Circulating MiRNA-335 in Breast Cancer Patients: A Prospective Study. *J Cell Biochem* **2018**, *120*, 8975–8982, doi:10.1002/jcb.28168.
9. Bakr, N.M.; Mahmoud, M.S.; Nabil, R.; Boushnak, H.; Swellam, M. Impact of Circulating MiRNA-373 on Breast Cancer Diagnosis through Targeting VEGF and Cyclin D1 Genes. *Journal of Genetic Engineering and Biotechnology* **2021**, *19*, 84, doi:10.1186/s43141-021-00174-7.
10. Zearo, S.; Kim, E.; Zhu, Y.; Zhao, J.T.; Sidhu, S.B.; Robinson, B.G.; Soon, P.S.H. MicroRNA-484 Is More Highly Expressed in Serum of Early Breast Cancer Patients Compared to Healthy Volunteers. *BMC Cancer* **2014**, *14*, 200, doi:10.1186/1471-2407-14-200.
11. Ali, S.A.; Abdulrahman, Z.F.A.; Faraidun, H.N. Circulatory MiRNA-155, MiRNA-21 Target PTEN Expression and Activity as a Factor in Breast Cancer Development. *Cell Mol Biol* **2020**, *66*, 44–50.
12. Li, Q.; Liu, M.; Ma, F.; Luo, Y.; Cai, R.; Wang, L.; Xu, N.; Xu, B. Circulating MiR-19a and MiR-205 in Serum May Predict the Sensitivity of Luminal a Subtype of Breast Cancer Patients to Neoadjuvant Chemotherapy with Epirubicin plus Paclitaxel. *PLoS One* **2014**, *9*, e104870, doi:10.1371/journal.pone.0104870.

13. Lopes, B.C.; Braga, C.Z.; Ventura, F. V.; de Oliveira, J.G.; Kato-Junior, E.M.; Bordin-Junior, N.A.; Zuccari, D.A.P.C. MiR-210 and MiR-152 as Biomarkers by Liquid Biopsy in Invasive Ductal Carcinoma. *J Pers Med* **2021**, *11*, 31, doi:10.3390/jpm11010031.
14. Shen, J.; Hu, Q.; Schrauder, M.; Yan, L.; Wang, D.; Medico, L.; Guo, Y.; Yao, S.; Zhu, Q.; Liu, B.; et al. Circulating MiR-148b and MiR-133a as Biomarkers for Breast Cancer Detection. *Oncotarget* **2014**, *5*, 5284–5294, doi:10.18632/oncotarget.2014.
15. Hesari, A.R.; Azizian, M.; Darabi, H.; Nesaei, A.; Hosseini, S.A.; Salarinia, R.; Motaghi, A.A.; Ghasemi, F. Expression of Circulating MiR-17, MiR-25, and MiR-133 in Breast Cancer Patients. *J Cell Biochem* **2019**, *120*, 7109–7114, doi:10.1002/jcb.27984.
16. Zeng, Z.; Chen, X.; Zhu, D.; Luo, Z.; Yang, M. Low Expression of Circulating MicroRNA-34c Is Associated with Poor Prognosis in Triple-Negative Breast Cancer. *Yonsei Med J* **2017**, *58*, 697–702, doi:10.3349/ymj.2017.58.4.697.
17. Heneghan, H.M.; Miller, N.; Lowery, A.J.; Sweeney, K.J.; Newell, J.; Kerin, M.J. Circulating MicroRNAs as Novel Minimally Invasive Biomarkers for Breast Cancer. *Ann Surg* **2010**, *251*, 499–505, doi:10.1097/SLA.0b013e3181cc939f.
18. McAnena, P.; Tanriverdi, K.; Curran, C.; Gilligan, K.; Freedman, J.E.; Brown, J.A.L.; Kerin, M.J. Circulating MicroRNAs MiR-331 and MiR-195 Differentiate Local Luminal a from Metastatic Breast Cancer. *BMC Cancer* **2019**, *19*, 436, doi:10.1186/s12885-019-5636-y.
19. Papadaki, C.; Stoupis, G.; Tsalikis, L.; Monastirioti, A.; Papadaki, M.; Maliotis, N.; Stratigos, M.; Mastrostamatis, G.; Mavroudis, D.; Agelaki, S. Circulating MiRNAs as a Marker of Metastatic Disease and Prognostic Factor in Metastatic Breast Cancer. *Oncotarget* **2019**, *10*, 966–981, doi:10.18632/oncotarget.26629.
20. Papadaki, C.; Stratigos, M.; Markakis, G.; Spiliotaki, M.; Mastrostamatis, G.; Nikolaou, C.; Mavroudis, D.; Agelaki, S. Circulating MicroRNAs in the Early Prediction of Disease Recurrence in Primary Breast Cancer. *Breast Cancer Research* **2018**, *20*, 72, doi:10.1186/s13058-018-1001-3.
21. Li, H.; Liu, J.; Chen, J.; Wang, H.; Yang, L.; Chen, F.; Fan, S.; Wang, J.; Shao, B.; Yin, D.; et al. A Serum MicroRNA Signature Predicts Trastuzumab Benefit in HER2-Positive Metastatic Breast Cancer Patients. *Nat Commun* **2018**, *9*, 1614, doi:10.1038/s41467-018-03537-w.
22. Sahlberg, K.K.; Bottai, G.; Naume, B.; Burwinkel, B.; Calin, G.A.; Børresen-Dale, A.-L.; Santarpia, L. A Serum MicroRNA Signature Predicts Tumor Relapse and Survival in Triple-Negative Breast Cancer Patients. *Clinical Cancer Research* **2015**, *21*, 1207–1214, doi:10.1158/1078-0432.CCR-14-2011.
23. Eichelser, C.; Flesch-Janys, D.; Chang-Claude, J.; Pantel, K.; Schwarzenbach, H. Deregulated Serum Concentrations of Circulating Cell-Free MicroRNAs MiR-17, MiR-34a, MiR-155, and MiR-373 in Human Breast Cancer Development and Progression. *Clin Chem* **2013**, *59*, 1489–1496, doi:10.1373/clinchem.2013.205161.
24. Li, M.; Zou, X.; Xia, T.; Wang, T.; Liu, P.; Zhou, X.; Wang, S.; Zhu, W. A Five-MiRNA Panel in Plasma Was Identified for Breast Cancer Diagnosis. *Cancer Med* **2019**, *8*, 7006–7017, doi:10.1002/cam4.2572.
25. Ibrahim, A.M.; Said, M.M.; Hilal, A.M.; Medhat, A.M.; Abd Elsalam, I.M. Candidate Circulating MicroRNAs as Potential Diagnostic and Predictive Biomarkers for the Monitoring of Locally Advanced Breast Cancer Patients. *Tumor Biology* **2020**, *42*, 1010428320963811, doi:10.1177/1010428320963811.

26. Shimomura, A.; Shiino, S.; Kawauchi, J.; Takizawa, S.; Sakamoto, H.; Matsuzaki, J.; Ono, M.; Takeshita, F.; Niida, S.; Shimizu, C.; et al. Novel Combination of Serum MicroRNA for Detecting Breast Cancer in the Early Stage. *Cancer Sci* **2016**, *107*, 326–334, doi:10.1111/cas.12880.
27. Matamala, N.; Vargas, M.T.; González-Cámpora, R.; Miñambres, R.; Arias, J.; Menéndez, P.; Andrés-León, E.; Mez-López, G.G.; Yanowsky, K.; Calvete-Candenas, J.; et al. Tumor MicroRNA Expression Profiling Identifies Circulating MicroRNAs for Early Breast Cancer Detection. *Clin Chem* **2015**, *61*, 1098–1106, doi:10.1373/clinchem.2015.238691.
28. Li, M.; Zhou, Y.; Xia, T.; Zhou, X.; Huang, Z.; Zhang, H.; Zhu, W.; Ding, Q.; Wang, S. Circulating MicroRNAs from the MiR-106a–363 Cluster on Chromosome X as Novel Diagnostic Biomarkers for Breast Cancer. *Breast Cancer Res Treat* **2018**, *170*, 257–270, doi:10.1007/s10549-018-4757-3.
29. Zhang, K.; Wang, Y.Y.; Xu, Y.; Zhang, L.; Zhu, J.; Si, P.C.; Wang, Y.W.; Ma, R. A Two-MiRNA Signature of Upregulated MiR-185-5p and MiR-362-5p as a Blood Biomarker for Breast Cancer. *Pathol Res Pract* **2021**, *222*, 153458, doi:10.1016/j.prp.2021.153458.
30. Huo, D.; Clayton, W.M.; Yoshimatsu, T.F.; Chen, J.; Olopade, O.I. Identification of a Circulating MicroRNA Signature to Distinguish Recurrence in Breast Cancer Patients. *Oncotarget* **2016**, *7*, 55231–55248, doi:10.18632/oncotarget.10485.
31. Qattan, A.; Al-Tweigeri, T.; Alkhayal, W.; Suleman, K.; Tulbah, A.; Amer, S. Clinical Identification of Dysregulated Circulating MicroRNAs and Their Implication in Drug Response in Triple Negative Breast Cancer (TNBC) by Target Gene Network and Meta-Analysis. *Genes (Basel)* **2021**, *12*, 549, doi:10.3390/genes12040549.
32. Cuk, K.; Zucknick, M.; Madhavan, D.; Schott, S.; Golatta, M.; Heil, J.; Marmé, F.; Turchinovich, A.; Sinn, P.; Sohn, C.; et al. Plasma MicroRNA Panel for Minimally Invasive Detection of Breast Cancer. *PLoS One* **2013**, *8*, e76729, doi:10.1371/journal.pone.0076729.
33. Satomi-Tsushita, N.; Shimomura, A.; Matsuzaki, J.; Yamamoto, Y.; Kawauchi, J.; Takizawa, S.; Aoki, Y.; Sakamoto, H.; Kato, K.; Shimizu, C.; et al. Serum MicroRNA-Based Prediction of Responsiveness to Eribulin in Metastatic Breast Cancer. *PLoS One* **2019**, *14*, e0222024, doi:10.1371/journal.pone.0222024.
34. Jang, J.Y.; Kim, Y.S.; Kang, K.N.; Kim, K.H.; Park, Y.J.; Kim, C.W. Multiple MicroRNAs as Biomarkers for Early Breast Cancer Diagnosis. *Mol Clin Oncol* **2021**, *14*, 31, doi:10.3892/mco.2020.2193.
35. Hamam, R.; Ali, A.M.; Alsaleh, K.A.; Kassem, M.; Alfayez, M.; Aldahmash, A.; Alajez, N.M. MicroRNA Expression Profiling on Individual Breast Cancer Patients Identifies Novel Panel of Circulating MicroRNA for Early Detection. *Sci Rep* **2016**, *6*, 25997, doi:10.1038/srep25997.
36. Khodadadi-Jamayran, A.; Akgol-Oksuz, B.; Afanasyeva, Y.; Heguy, A.; Thompson, M.; Ray, K.; Giro-Perafita, A.; Sánchez, I.; Wu, X.; Tripathy, D.; et al. Prognostic Role of Elevated Mir-24-3p in Breast Cancer and Its Association with the Metastatic Process. *Oncotarget* **2018**, *9*, 12868–12878, doi:10.18632/oncotarget.24403.
37. Yoshikawa, M.; Iinuma, H.; Umemoto, Y.; Yanagisawa, T.; Matsumoto, A.; Jinno, H. Exosome-Encapsulated MicroRNA-223-3p as a Minimally Invasive Biomarker for the Early Detection of Invasive Breast Cancer. *Oncol Lett* **2018**, *15*, 9584–9592, doi:10.3892/ol.2018.8457.
38. Wang, X.; Qian, T.; Bao, S.; Zhao, H.; Chen, H.; Xing, Z.; Li, Y.; Zhang, M.; Meng, X.; Wang, C.; et al. Circulating Exosomal MiR-363-5p Inhibits Lymph Node Metastasis by Downregulating

PDGFB and Serves as a Potential Noninvasive Biomarker for Breast Cancer. *Mol Oncol* **2021**, *15*, 2466–2479, doi:10.1002/1878-0261.13029.
39. Moloney, B.M.; Gilligan, K.E.; Joyce, D.P.; O'Neill, C.P.; O'Brien, K.P.; Khan, S.; Glynn, C.L.; Waldron, R.M.; Maguire, C.M.; Holian, E.; et al. Investigating the Potential and Pitfalls of EV-Encapsulated MicroRNAs as Circulating Biomarkers of Breast Cancer. *Cells* **2020**, *9*, 141, doi:10.3390/cells9010141.
40. Zheng, X.; Lu, S.; He, Z.; Huang, H.; Yao, Z.; Miao, Y.; Cai, C.; Zou, F. MCU-Dependent Negative Sorting of MiR-4488 to Extracellular Vesicles Enhances Angiogenesis and Promotes Breast Cancer Metastatic Colonization. *Oncogene* **2020**, *39*, 6975–6989, doi:10.1038/s41388-020-01514-6.
41. Zhang, G.; Zhang, W.; Li, B.; Stringer-Reasor, E.; Chu, C.; Sun, L.; Bae, S.; Chen, D.; Wei, S.; Jiao, K.; et al. MicroRNA-200c and MicroRNA-141 Are Regulated by a FOXP3-KAT2B Axis and Associated with Tumor Metastasis in Breast Cancer. *Breast Cancer Research* **2017**, *19*, 73, doi:10.1186/s13058-017-0858-x.
42. Hannafon, B.N.; Trigoso, Y.D.; Calloway, C.L.; Zhao, Y.D.; Lum, D.H.; Welm, A.L.; Zhao, Z.J.; Blick, K.E.; Dooley, W.C.; Ding, W.Q. Plasma Exosome MicroRNAs Are Indicative of Breast Cancer. *Breast Cancer Research* **2016**, *18*, 90, doi:10.1186/s13058-016-0753-x.
43. Zhang, Z.; Zhang, L.; Yu, G.; Sun, Z.; Wang, T.; Tian, X.; Duan, X.; Zhang, C. Exosomal MiR-1246 and MiR-155 as Predictive and Prognostic Biomarkers for Trastuzumab-Based Therapy Resistance in HER2-Positive Breast Cancer. *Cancer Chemother Pharmacol* **2020**, *86*, 761–772, doi:10.1007/s00280-020-04168-z.
44. Rodríguez-Martínez, A.; De Miguel-Pérez, D.; Ortega, F.G.; García-Puche, J.L.; Robles-Fernández, I.; Exposito, J.; Martorell-Marugan, J.; Carmona-Sáez, P.; Garrido-Navas, M.D.C.; Rolfo, C.; et al. Exosomal MiRNA Profile as Complementary Tool in the Diagnostic and Prediction of Treatment Response in Localized Breast Cancer under Neoadjuvant Chemotherapy. *Breast Cancer Research* **2019**, *21*, 21, doi:10.1186/s13058-019-1109-0.
45. Wu, H.; Wang, Q.; Zhong, H.; Li, L.; Zhang, Q.; Huang, Q.; Yu, Z. Differentially Expressed MicroRNAs in Exosomes of Patients with Breast Cancer Revealed by Next-Generation Sequencing. *Oncol Rep* **2020**, *43*, 240–250, doi:10.3892/or.2019.7401.
46. Koi, Y.; Tsutani, Y.; Nishiyama, Y.; Ueda, D.; Ibuki, Y.; Sasada, S.; Akita, T.; Masumoto, N.; Kadoya, T.; Yamamoto, Y.; et al. Predicting the Presence of Breast Cancer Using Circulating Small RNAs, Including Those in the Extracellular Vesicles. *Cancer Sci* **2020**, *111*, 2104–2115, doi:10.1111/cas.14393.
47. Ahmed, S.H.; Espinoza-Sánchez, N.A.; El-Damen, A.; Fahim, S.A.; Badawy, M.A.; Greve, B.; El-Shinawi, M.; Götte, M.; Ibrahim, S.A. Small Extracellular Vesicle-Encapsulated MiR-181b-5p, MiR-222-3p and Let-7a-5p: Next Generation Plasma Biopsy-Based Diagnostic Biomarkers for Inflammatory Breast Cancer. *PLoS One* **2021**, *16*, e0250642, doi:10.1371/journal.pone.0250642.
48. Chen, W.; Cao, R.; Su, W.; Zhang, X.; Xu, Y.; Wang, P.; Gan, Z.; Xie, Y.; Li, H.; Qin, J. Simple and Fast Isolation of Circulating Exosomes with a Chitosan Modified Shuttle Flow Microchip for Breast Cancer Diagnosis. *Lab Chip* **2021**, *21*, 1759–1770, doi:10.1039/d0lc01311k.
49. Ni, Q.; Stevic, I.; Pan, C.; Müller, V.; Oliviera-Ferrer, L.; Pantel, K.; Schwarzenbach, H. Different Signatures of MiR-16, MiR-30b and MiR-93 in Exosomes from Breast Cancer and DCIS Patients. *Sci Rep* **2018**, *8*, 12974, doi:10.1038/s41598-018-31108-y.

50. Ozawa, P.M.M.; Vieira, E.; Lemos, D.S.; Souza, I.L.M.; Zanata, S.M.; Pankievicz, V.C.; Tuleski, T.R.; Souza, E.M.; Wowk, P.F.; Urban, C.D.A.; et al. Identification of MiRNAs Enriched in Extracellular Vesicles Derived from Serum Samples of Breast Cancer Patients. *Biomolecules* **2020**, *10*, 150, doi:10.3390/biom10010150.
51. Sueta, A.; Fujiki, Y.; Goto-Yamaguchi, L.; Tomiguchi, M.; Yamamoto-Ibusuki, M.; Iwase, H.; Yamamoto, Y. Exosomal MiRNA Profiles of Triple-negative Breast Cancer in Neoadjuvant Treatment. *Oncol Lett* **2021**, *22*, 819, doi:10.3892/ol.2021.13080.
52. Todorova, V.K.; Byrum, S.D.; Gies, A.J.; Haynie, C.; Smith, H.; Reyna, N.S.; Makhoul, I. Circulating Exosomal MicroRNAs as Predictive Biomarkers of Neoadjuvant Chemotherapy Response in Breast Cancer. *Current Oncology* **2022**, *29*, 613–630, doi:10.3390/curroncol29020055.
53. Sueta, A.; Yamamoto, Y.; Tomiguchi, M.; Takeshita, T.; Yamamoto-Ibusuki, M.; Iwase, H. Differential Expression of Exosomal MiRNAs between Breast Cancer Patients with and without Recurrence. *Oncotarget* **2017**, *8*, 69934–69944, doi:10.18632/oncotarget.19482.
54. Stevic, I.; Müller, V.; Weber, K.; Fasching, P.A.; Karn, T.; Marmé, F.; Schem, C.; Stickeler, E.; Denkert, C.; Van Mackelenbergh, M.; et al. Specific MicroRNA Signatures in Exosomes of Triple-Negative and HER2-Positive Breast Cancer Patients Undergoing Neoadjuvant Therapy within the GeparSixto Trial. *BMC Med* **2018**, *16*, 179, doi:10.1186/s12916-018-1163-y.
